# Tracheotomy in Tonsilitis

**Published:** 1838

**Authors:** James Lyman

**Affiliations:** Austinburg, Ohio


### Art. V.—Tracheotomy in Tonsilitis.

*A case of Tronsilitis requiring, and successfully treated, by Tracheotomy.* By Dr. James Lyman, of Austinburg, Ohio.

Mrs. L——, the subject of this case was 23 years of age, of a nervous temperament, but generally healthy. Sudden changes in the weather had been frequent, and on going to church the 5th of March, 1836, she wet her feet, and returned home with her stockings wet and frozen. She however suffered no particular inconvenience, until the morning of the 7th, when she woke with soreness and pain in the fauces, redness and enlargement of the tonsils, together with some degree of lassitude and headache. Detergent gargles were used during the day, but she continued to grow worse. In the evening, I prescribed a cathartic of calomel and sulphate of magnesia, and applied a blister to the neck. On the morning of the 8th, the cathartic had operated copiously, and the blister filled well; but no relief had been obtained. Pulse nearly natural, or a little depressed; deglutition very difficult; respiration somewhat impeded; skin nearly of the natural temperature. Gargles continued, and diaphoretics administered, as well as the case would admit. In the evening, all the symptoms more aggravated; fever more apparent; skin hot and dry; pulse somewhat excited; pain, to use a common expression, in the bones, and sense of pressure in the head: bled from two orifices, until syncope ensued, which afforded considerable relief for a few hours, when the pain returned with more violence than ever; deglutition completely stopped by the morning of the 9th, and respiration more difficult; no rest since she was taken. Scarified the tonsils, and obtained about an ounce of blood, which gave temporary relief. Another blister was applied to the upper part of the chest, which filled well in a few hours, and occasioned a severe strangury, that lasted six or eight hours, and whilst this continued, very little pain was complained of in the throat, and the respiration was freer; but all the symptoms returned with aggravated violence as the strangury subsided. On the 20th, respiration more difficult; the patient's tongue protruded from the mouth; eyes swollen and projecting; more pain throughout the system; skin hotter and dryer; pulse so much increased in strength and frequency, that I thought it best to take more blood, which was suffered to flow from two orifices, until faintness approached. This once more gave some relief from pain; but the respiration became more and more difficult, so that, on the morning of the 11th, the eyes were much strained out of their sockets, appeared almost immoveable, and she was blind. The throat was livid, and nearly insensible to a strong decoction of cayenne pepper, which was then used to wet the fauces.

Such was her situation—fast failing for the want of breath, liable to serious lesion of the brain, in consequence of the violent distension of its blood-vessels, and the swelling of the throat still increasing, when I came to the determination to operate on the trachea. Counsel had been sent for, but none obtained. I therefore determined to operate alone. The patient was placed in an arm chair, with a pillow to her shoulders, and inclined backwards so as to render the trachea as prominent as possible. A transverse incision was then made, immediately over the interstice, through the integuments, between the first and second rings of the trachea, and the rings then divided with a bistoury. The thyroid gland was wholly avoided, and no vessel of importance injured; of course but little bleeding ensued. A leaden tube, made by myself at the moment, was then introduced, and fastened by passing some ligatures around the neck. The patient was then placed in a recumbent position, where, being at liberty to breathe with freedom, she became much revived. Her eyes by degrees resumed their wonted position in the sockets, and her countenance lost its bloated and purple aspect. A strong decoction of cayenne pepper was continued as a wash for the parts. On the 12th, intumescence somewhat greater that on the 11th, but not pointed so as to afford any chance for opening it; fauces exceedingly black and fœtid; some delirium; pepper continued, and nourishment and stimlants thrown up the bowels by means of a stomach pump. On the 13th, symptoms and treatment the same as on the 12th, until about midnight, when the tumour burst whilst she was in a sitting posture, and at once her lungs were filled with pus. I was in an adjoining room, and was immediately called. I entered the room, and found her, contrary to my wishes, in a sitting attitude, in which she had been placed, for the purpose of rendering respiration more easy. I immediately seized hold of her, and placed her in a situation where her head and chest should form the lowest part of her body. No signs were now to be seen of respiration, excepting an *occasional* gasp, and animation seemed nearly suspended. I now caught hold of a teaspoon-full of aqua ammoniæ, and dashed it into her mouth, which produced an effort to cough. This was somewhat successful, and threw off a part of the load from the lungs. By repeated efforts of this kind, the lungs were unloaded, and respiration fully re-established. From this time she gradually improved, though large pieces sloughed away from her throat, and the incision into the trachea healed without the slightest difficulty. In dressing the wound, care was taken to embrace all the integuments within the ligature, down to the rings of the trachea, and one stitch alone was all that was required.

Austinburg, October 1837.

